# Unraveling the Mode of Action of *Cordyceps fumosorosea*: Potential Biocontrol Agent against *Plutella xylostella* (Lepidoptera: Plutellidae)

**DOI:** 10.3390/insects12020179

**Published:** 2021-02-18

**Authors:** Yanyuan Lei, Abid Hussain, Zhaoying Guan, Desen Wang, Waqar Jaleel, Lihua Lyu, Yurong He

**Affiliations:** 1 Guangdong Provincial Key Laboratory of High Technology for Plant Protection, Plant Protection Research Institute, Guangdong Academy of Agricultural Sciences, Guangzhou 510640, China; leiyanyuan@gdaas.cn (Y.L.); waqar4me@yahoo.com (W.J.); lhlu@gdppri.com (L.L.); 2 Institute of Research and Consultancy, King Faisal University, Hofuf 31982, Saudi Arabia; abhussain@kfu.edu.sa; 3 Ministry of Environment, Water and Agriculture, Riyadh 11442, Saudi Arabia; 4 School of Applied Biology, Shenzhen Institute of Technology, Shenzhen 518116, China; guanzhaoying717@126.com; 5 Department of Entomology, South China Agricultural University, Guangzhou 510642, China; desen@scau.edu.cn; 6 Key Laboratory of Bio-Pesticide Innovation and Application, Guangzhou 510642, China; 7 Engineering Research Center of Biological Control, Ministry of Education, Guangzhou 510642, China

**Keywords:** *Cordyceps fumosorosea*, diamondback moth, SEM, electron microscopy, cuticle, conidium, infection process, cuticle topography, appressorium

## Abstract

**Simple Summary:**

The diamondback moth is a major destructive pest of cruciferous plants with a worldwide global distribution. Biological control, especially the use of insect pathogenic fungi as a novel control measure, remains the subject of intensive experimentation. The goal is to develop an efficient and eco-friendly alternative to chemical insecticides. The current study successfully explored and compared in-depth infection events between highly pathogenic and less pathogenic fungal strains. Our study for the first time provides new insights into the mechanisms by which fungal conidia parasitize diamondback moth larvae and ultimately aid in the development of the highly pathogenic strain into an effective biopesticide for the eco-friendly management of the diamondback moth.

**Abstract:**

The entomopathogenic fungus, *Cordyceps fumosorosea* is a potential eco-friendly biocontrol agent. The present study revealed the entire course of infection of *P. xylostella* by *C. fumosorosea* with particular reference to cuticular penetration. Comparative studies on the infection of *Plutella xylostella* larvae by two strains of *C. fumosorosea* with different pathogenicity were carried out using light, scanning, and transmission electron microscopy. We found that *C. fumosorosea* tended to adhere to the cuticle surfaces containing protrusions. Although conidia of the lower pathogenic strain IFCF-D58 germinated, they failed to penetrate and complete the development cycle. In contrast, the higher pathogenic strain IFCF01 began to germinate within 4 h and attached to the cuticle by a thin mucilaginous matrix within 8 h post-inoculation. After 24 h post-inoculation, germ tubes and penetrating hyphae reached the cuticular epidermis and began to enter the haemocoel. Within 36 h post-inoculation, the hyphal bodies colonized the body cavity. Hyphae penetrated from inside to outside of the body after 48 h and sporulated the cadavers. After 72 h post-inoculation, numerous conidia emerged and the mycelial covered the entire cuticular surface. The two strains showed similarities in terms of conidial size and germination rate. However, IFCF-D58 exhibited significantly fewer appressoria and longer penetrating hyphae compared to the more infective IFCF01 on all surface topographies. The current pathogen invasion sequence of events suggested that the aggressive growth and propagation along with rapid and massive in vivo production of blastospores facilitate the conidia of IFCF01 to quickly overcome the diamondback moth’s defense mechanism.

## 1. Introduction

The diamondback moth, *Plutella xylostella* (Lepidoptera: Plutellidae) is a major destructive pest of cruciferous plants worldwide [1]. Current control methods are based on the frequent application of chemical insecticides [2]. However, the populations of *P. xylostella* have shown resistance to synthetic insecticides in certain parts of the world [3,4,5]. In order to protect the ecosystem, biological control agents as a novel measure for these agricultural pests could be an efficient alternative to chemical insecticides. Among the various biocontrol strategies commonly practiced against agricultural pests, the use of entomopathogenic fungi is an efficient and environmentally favorable biocontrol agent-based pest management method [6,7,8,9]. Some entomopathogenic fungi (e.g., *Beauveria* sp., *Metarhizium* sp.) are recognized as primary agents to control this pest [7], and among other fungal pathogens, *Cordyceps fumosorosea* (Wize) (formerly *Isaria fumosorosea* [10] (Hypocreales: Cordycipitaceae) also reported to infect numerous pest species [11], including diamondback moths [12,13,14], termites [15], whiteflies [16,17], Asian citrus psyllids [18,19,20], red palm weevils [21], and some other agricultural pests [22,23].

Unlike other control agents that need to be ingested, fungi infect target hosts by cuticular penetration, and overcome the target host’s defense mechanism independent of their growth habits. Their mode of action against host insects begins with the attachment of conidia to cuticles, followed by germination and cuticle penetration. Once through the cuticle, the conidia can access the host hemolymph and develop into yeast-like hyphal bodies (blastospores). They then propagate in the hemocoel where they combat host cellular and humoral immune defense mechanisms. In order to counter fungal conidia, immunologically activated hemocytes defend against these pathogens through cellular processes such as phagocytosis, encapsulation, and nodulation [24,25,26]. The humoral defense responses rely on specific signal transduction pathways modulated by particular classes of pathogen recognition receptors that are mainly responsible for the detection of invading pathogens [27,28,29,30]. As a consequence of long-term co-evolution, entomopathogens have reprogrammed their virulence mechanism to overcome host defense mechanism [31,32,33].

Numerous efforts have been devoted to understanding infection processes using *B. bassiana* and *M. anisopliae*. Host death is due to mycotoxin production (e.g., beauvericin, destruxin) [34,35,36] by the invading fungal pathogen. Some bioactive molecules of *C. fumosorosea* have recently been identified [19,37], but it is still unclear whether the modes of action of *C. fumosorosea* are the same with toxin-secreting fungi such as *B. bassiana* and *M. anisopliae*. The current state of knowledge suggests that the penetration, colonization, and conidiogenesis phases of conidia are related to the faster rate of insect mortality [38]. Given the numerous potential explanations for the differences in pathogenicity among fungal strains, a comparison of the infection behavior of strains against *P. xylostella* helped to identify the most likely explanation for the virulence differences [39]. However, little information is available on the pathology of *C. fumosorosea*. For these reasons, clarifying the infection processes of *C. fumosorosea* when used against *P. xylostella* will allow us to better understand its mode of action to combat the target host’s defense mechanism.

In our previous study, a strain of *C. fumosorosea* SCAU-IFCF01 (IFCF01) demonstrated high pathogenicity against *P. xylostella* under laboratory conditions (the median lethal time (LT_50_) for the 2nd-instar larvae was 1.72 d at a concentration of 1 × 10^7^ conidia/mL) [12]. This was the first time such a rapid mortality against *P. xylostella* was reported [12,13,40,41,42], which thus suggests significant potential for wider applications in the biocontrol field. In our previous study, we provided an overview of infection symptoms, germination, attachment, penetration and conidial reproduction, and pathological changes of *C. fumosorosea* IFCF01 and the low pathogenicity derivative isolate IFCF-D58 towards *P. xylostella* [12,13,40]. However, the factors and underlying mechanisms determining host specificity and high pathogenicity of the strain IFCF01 are poorly understood. The current study is aimed to describe the external development cycle of *C. fumosorosea* IFCF01 on *P. xylostella* larvae to identify the stages of infection at which the less pathogenic strain (IFCF-D58) is deficient, and to investigate whether the infection behavior of *C. fumosorosea* conidia on the surface of the cuticle could explain the pathogenicity of the strains. This study will further provide new insights into the mechanisms by which *C. fumosorosea* parasitizes *P. xylostella* and ultimately aids in the development of this highly pathogenic strain into an effective biopesticide for the eco-friendly management of the diamondback moth.

## 2. Materials and Methods

### 2.1. Cordyceps fumosorosea

Cultures of *Cordyceps fumosorosea* strain IFCF01 (formerly EBCL03011) were originally derived from cadavers of naturally infected Formosan subterranean termites, *Coptotermes formosanus* (Shiraki) [12], and deposited at the China Center for Type Culture Collection, Wuhan, China, under the accession number M 2013526. The second strain, IFCF-D58, was obtained by successive sub-culturing, more than 30 times, of the highly virulent strain IFCF01, and deposited at the Engineering Research Center of Biological Control, South China Agricultural University, China, under accession number IFCF-D58. Our previous study showed that the *C. fumosorosea* strain IFCF01 was able to kill diamondback moth larvae quicker than strain IFCF-D58 [13], and the reduced infectivity of IFCF-D58 against *P. xylostella* larvae could not be restored by passing through its host [14]. All fungal strains were grown at 25 °C in darkness on PDA for 20 d. A 0.5% (*v*/*v*) Tween-80 solution was used to harvest the 20-day-old culture to prepare a conidial suspension with a concentration of 1 × 10^8^ conidia/mL by using a hemocytometer [40].

### 2.2. Plutella Xylostella Larvae

Larvae and pupae of *P. xylostella* were collected from Baiyun in Guangzhou City, Guangdong Province, China (23°06′ N, 112°12′ E), and maintained on Chinese cabbage (*Brassica oleracea* L., cv. Yueshu) without insecticide exposure for more than five generations. Adults were fed with a 25% honey solution. The laboratory population was maintained at 26 ± 1 °C, and 70 ± 10% RH under 12:12 h light:dark cycle laboratory conditions. The 4th-instar larvae were collected to await further processing.

### 2.3. Inoculation of P. xylostella with C. fumosorosea

Ninety 4th instar larvae for each microscopic analysis were used for inoculation using the conidial suspension of *C. fumosorosea* strain IFCF01 (1 × 10^8^ conidia/mL), *C. fumosorosea* strain IFCF-D58 (1 × 10^8^ conidia/mL) and control (conidia-free solution of 0.5% Tween-80) by immersion for 10 sec in their respective solutions. Following inoculation, the diamondback back moth larvae, whether immersed in the conidial suspension of *C. fumosorosea* strain IFCF01, *C. fumosorosea* strain IFCF-D58, or control, were separately placed in 90 mm diameter Petri dishes provided with filter paper moistened with sterile distilled water, and provided with cabbage leaves for food, which were replaced every day. Petri dishes were maintained in an incubator under controlled conditions mentioned in Section 2.1. A total of three replicates per strain were prepared for subsequent study.

### 2.4. Observation of Larval Appearance under Light Microscopy (LM)

The immersed 4th instar diamondback moth larvae were fed on cabbage leaves for different time intervals including 8, 16, 24, 48, and 72 h post-inoculation. Each studied time interval, six 4th instar diamondback moth larvae that represent six replicates were used to observe the changes in larval appearance, exposed to conidial suspension of the studied strains. A Keyence VHX-S550-(D) digital microscope (Keyence Corporation, Osaka, Japan) was used to capture photographs of the specimens in order to analyze the changes of the larval cuticles of *P. xylostella* following inoculation with *C. fumosorosea* strains.

### 2.5. Scanning Electron Microscopy (SEM)

Larval cuticles were observed by scanning electron microscopy at 4, 8, 16, 24, 36, 48, 60, and 72 h post-inoculation to document the development of IFCF01 and IFCF-D58 conidia. Three larvae (three replicates) for each selected time interval were observed for SEM analysis. The glutaraldehyde solution at 4% (*v*/*v*) strength in 0.1M cacodylate buffer was used to fix larval samples for 12 h at 4 °C. The specimens were then dehydrated for 10 min each in a series of ascending order prepared serial dilutions of ethanol (50, 70, 80, 90, and 100%). After dehydration, the specimens were subsequently dipped for 30 min in isoamyl acetate. The specimens were dried with a critical-point drying apparatus using liquid carbon dioxide. After mounting the specimens on the microscope slides, a gold palladium film was used to coat the specimens for subsequent examination and the specimens were photographed at 15 kV under an environmental scanning electron microscope (Philips XL-30 ESEM). Each replicate (larva) was thoroughly observed under SEM, and each replicate microscopic observation was recorded by observing under different SEM 10 fields of vision.

### 2.6. Transmission Electron Microscopy (TEM)

Larvae that were to be examined with TEM were collected at 16, 24, 36, and 48 h after inoculation with IFCF01. Each time interval, six specimens per treatment were fixed as described in Section 2.4. After the fixation step, specimens were then post-fixed for 3 h at 4 °C using osmium tetroxide at a strength of 1% (*v*/*v*) prepared in phosphate buffer (0.1 M) with a pH of 7.2. The specimens were then stepwise dehydrated in a series of ethanol dilutions from 10 to 100%. After dehydration, specimens were then embedded using the embedding resin Epon 812. After embedding, semi-thin sections (1 µM) were stained using 1% (*v*/*v*) toluidine blue for subsequent microscopic examination under a Zeiss Primo Star light microscope. A Leica UCT ultra-microtome was used to cut semi-thin sections into ultrathin sections (0.08 µM) for counter staining (uranyl acetate and lead citrate) to examine specimens at 80 kV under a transmission electron microscope (FEI TECNAI-12 of FELMI-ZFE).

### 2.7. Conidial Growth Parameter Analysis on Diamondback Moth Larval Cuticle

To compare conidial infection patterns between strains IFCF01 and IFCF-D58, the size of conidia of both strains was measured by scanning their length and width under SEM at 4 h after inoculation. At least 60 conidia were counted per strain to determine conidia size. The proportion of conidia that had germinated at 16 h after inoculation and the proportion of appressoria relative to the total number of conidia were assessed, as was the length of the penetrating hyphae at 24 h after inoculation. For the determination of percentage germination and appressoria, 25 conidia at each surface topography were considered, with three larvae per strain treatment. The acanthoid surface topography includes the dorsum of the segments, and the thoracic and abdominal pleuron, excluding the setae and spiracle sites. For the estimation of the penetrating hyphae length, random counts were made for at least three to five conidia at each surface topography, from three larvae per strain treatment. Each surface topography of strain IFCF01 contained 15 conidia, while strain IFCF-D58 contained 10 conidia. A conidium with a germ tube equal to its width was considered to have germinated [43]. When the diameter of the distal end of the germ tube expands at least 1.5 times more than the width of the germ tube, it is considered to develop an appresorium. The length of the penetrating hyphae was defined as the length from germination to the penetration point. Prior to analysis, percentage data were transformed by arcsine square root. In order to gauge the differences in the conidial growth parameters, one-way analysis of variance (ANOVA) was applied, and significant differences among the means were computed by Tukey’s HSD test (α = 0.05) using SPSS version 22.0. (IBM Corp, Armonk, NY, USA)

## 3. Results

### 3.1. External Symptoms of P. xylostella Larvae Infected with C. fumosorosea

**Figure 1:**
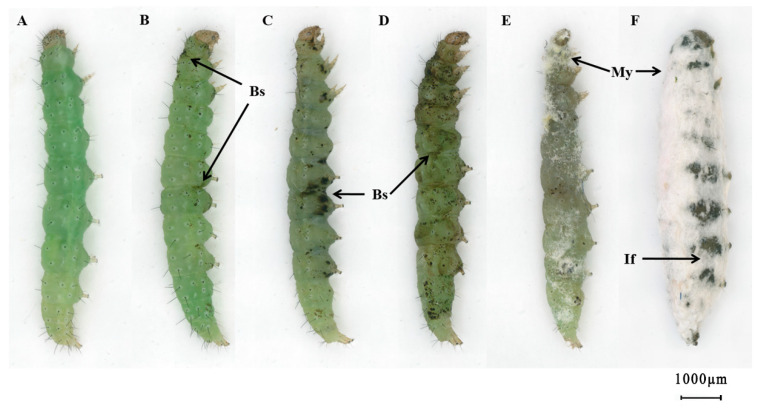
The external symptoms of *Plutella xylostella* larvae exposed to *Cordyceps fumosorosea* strain IFCF01, observed under a stereoscope. (**A**) Control group larva; (**B**) black spots (Bs) appeared on the larval cuticle at 8 h post-exposure; dark spots appeared on the entire body of diamondback moth larvae after 16 h (**C**) and 24 h (**D**) post-exposure; (**E**) mycelia (My) develop on the dead larval cadaver after 48 h post-exposure, while intersegmental folds (If) showed dense mycelial mass; (**F**) mycelia entirely covered the cadaver after 72 h post-exposure.

Diamondback moth larvae exhibited dysphoria following exposure with the pathogenic fungus *C. fumosorosea* IFCF01. Appressoria and invasion positions exhibited certain regularity. Intersegmental folds between the thoracic and abdominal segments, legs, and spiracles were the first invasion positions and a small area of melanotic encapsulation presented on the cuticle in the early stage of invasion (Figure 1B). As infection progressed, melanotic encapsulation on the abdominal segments showed comprehensive proliferation, from abdominal pleuron spread to the whole thoracic and abdominal pleuron; the level of melanotic encapsulation on spiracles and setae further deepened (Figure 1C,D). Finally, on the surfaces exhibiting melanotic encapsulation, hyphae penetrated integuments from outside to inside and then back outside, which rendered the infected larvae completely immobile and on the brink of death. After larval death, a change in the body color from light green (Figure 1A) to dark green (Figure 1D) was observed. Conidia, germination tubes, and hyphae developed on the cuticle and formed dense mycelium (Figure 1E,F).

### 3.2. Cuticle Topography of P. xylostella Larvae

**Figure 2:**
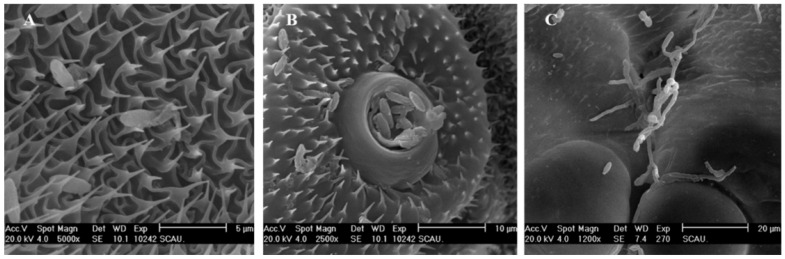
Cuticle topography of *Plutella xylostella* larvae with conidial adhesion. (**A**) Acanthoid surface topography; (**B**) Spinous surface topography; (**C**) Gentle surface topography.

The larval cuticle was classified according to its surface topography, which was described as an acanthoid, spinous, or gentle surface topography (Figure 2). These parts were covered with densely acanthoid protuberances; the cross-connections at the base of the acanthae were without perspicuous demarcation (Figure 2A). The spinous surface topography includes the center of the segments, sites beside the setae and spiracles, thoracic legs, and prolegs. The spinous surface topography exhibited regularly spinous protuberance, with clear separation and a flat part at the base of the spinous protuberances (Figure 2B). The gentle surface topography includes the head capsule, setal alveolus, spiracles, and segmental membrane. The surface of these sites was flat and smooth, without acanthoid or spinous protuberance (Figure 2C).

### 3.3. Fungal Attachment to the Insect Body

**Figure 3:**
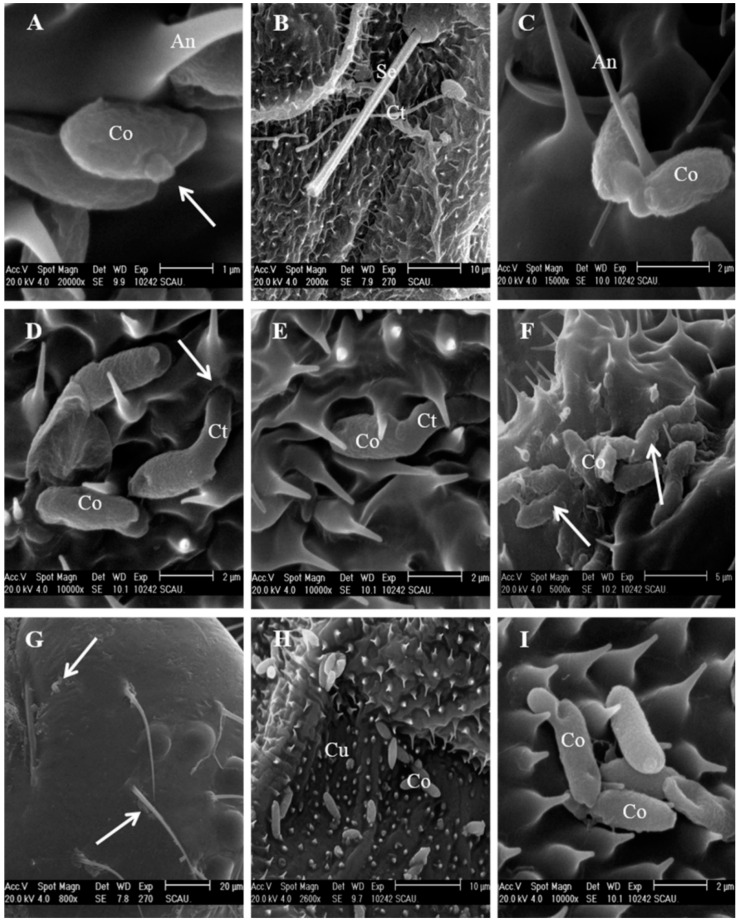
Conidial attachment and germination of *Cordyceps fumosorosea* on the cuticle of *Plutella xylostella* larvae observed under scanning electron microscope. A-F: IFCF01 strain; (**A**) A conidium (Co) attached to the abdominal pleuron surface and germinated to form a germ tube (arrow) at 4 h post-inoculation; An = antennae; (**B**) A pair of germ tubes formed from conidia at 16 h post-inoculation. Gt = germ tube, Se = seta; (**C**) Appressorium formation; (**D**) Conidia (Co) germinate, and the germ tube (Gt) penetrate (arrow) into the integument at the abdominal pleuron; (**E**) Germ-tube grew directionally along the base of the acanthae at the abdominal leg; (**F**) Appressoria covered with mucilage (arrows) at 8 h post-exposure. (**G**–**I**): IFCF-D58 strain; (**G**) Very few attached conidia (arrows) showing germination to the head capsule at 8 h post-inoculation; (**H**) Conidia (Co) adhered to the thoracic pleuron at 8 h post-inoculation. Cu = cuticle; (**I**) Conidia (Co) attach to the thoracic leg and germinate to form a germ tube at 16 h post-inoculation.

Conidia of *C. fumosorosea* IFCF01 attached firmly to all surface topographies of the cuticle, with a preference for surfaces containing protrusions (Figure 3). Conidia were observed in high densities mostly in the acanthoid and spinous surface topographies (Figure 2A,B), and less in the gentle surface topography (Figure 2C). The larval head capsule was strongly skeletonized, while the exoskeleton fused into a glazed, rigid capsule to which few conidia of IFCF01 were attached (Figure 2C). Some conidia were observed in the peritreme socket of the spiracles that inserted laterally on the thoracic and abdominal segments (Figure 2B).

Conidia of the IFCF01 strain germinated within 4 h following inoculation (Figure 3A). The slow cuticle surface contact might result in longer germ-tube growth (Figure 3B). Two types of fungal activity might occur during germination. Firstly, the distal tip of the germ tubes of the conidia might produce a specialized infection peg (Figure 3C), which actually penetrates through the cuticle of the host (Figure 3D). Secondly, germ tubes of the invading conidia might first develop hyphae, and then subsequently extend over the integument until a suitable invasion site is located, and a specialized infection peg at the hyphal tips is formed, which then penetrates through the cuticle of the host (Figure 3E). A mucilagenous coat was seen along the conidia and appressoria (Figure 3F).

None of the conidia of IFCF-D58 were found on the head capsule (Figure 3G). Germinated conidia were observed between the acanthae, but they were not able to develop further to reach at primary appressorium stage (Figure 3H). After germination, only a germ-tube close to the cuticle surface emerged at the periphery of the papilla (Figure 3I). After a short period of conidial growth, each germ-tube differentiated into a multi-lobed appressorium.

### 3.4. Fungal Penetration through Integument

**Figure 4:**
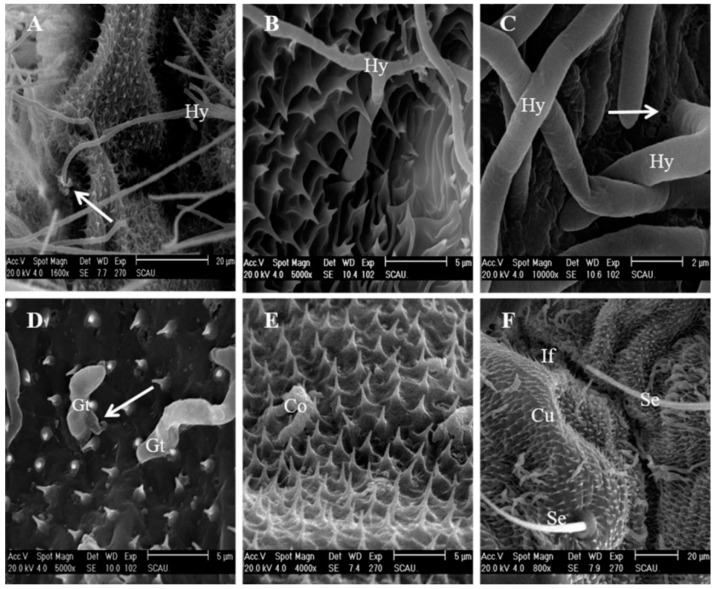
SEM photographs of penetration of *Cordyceps fumosorosea* on the surface of *Plutella xylostella* larvae. (A–C) IFCF01; (**A**) A hypha (Hy) was visible at an intersegmental fold piercing through an obvious invasion site (arrow); (**B**) Hyphae (Hy) extended over the cuticular surface (Cu) and invaded by penetration peg (Peg); (**C**) Hypha penetrated at 48 h after inoculation, the penetration site was marked with an arrow; (**D**–**F**) IFCF-D58; (**D**) Germ-tube (Gt) penetrated through the integument (arrow) at 24 h after inoculation; (**E**) The surface of ungerminated, non-viable conidia was strongly shriveled at the thoracic dorsal; (**F**) The intersegmental fold (If), setae (Se), and large numbers of acanthae are shown in the cuticle (Cu); Malformed hyphae on the cuticle and around the seta (Se), 36 h after inoculation.

As further deepened dark spots were detected on the cuticle after 24 h post-inoculation with IFCF01, it is reasonable to assume that the penetration period had largely occurred by that time. As the hyphae grew, a second or third generation of hyphae developed next to the initial appressorium (Figure 4A,B). Penetration holes were observed by hyphae invasion sites after 24 h (Figure 4A). Hyphae were found to penetrate from the inside to the outside of the body at 48 h (Figure 4C).

Penetration sites were rarely seen on the cuticle, except for a few individual conidia with penetration pegs, which were observed after 24 h post-inoculation with IFCF-D58 (Figure 4D). The primary appressorium was frequently malformed (Figure 4E,F). The surface of the ungerminated and non-viable conidium was distinctly shriveled compared to germinated conidium (Figure 4E). Germinated conidia showed malformed appressoria (Figure 4F). Cuticles did not inhibit germination and initial hyphal growth, but prevented hyphal development and caused growth distortion of *C. fumosorosea* IFCF-D58.

### 3.5. Physiological Parameters of the Two C. fumosorosea Strains with Different Pathogenicity

**Figure 5:**
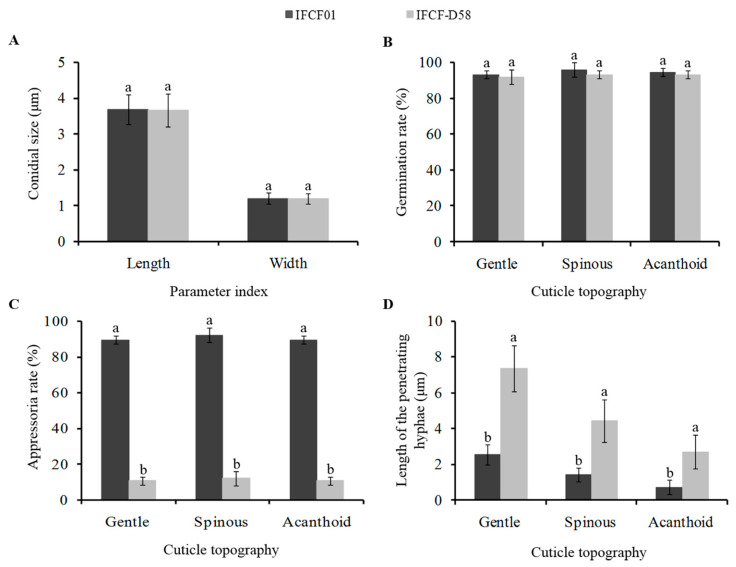
Comparison of conidial growth parameters of *Cordyceps fumosorosea* strains IFCF01 and IFCF-D58 on different cuticle topographies of *Plutella xylostella* larvae. (**A**) Conidia size, (**B**) Germination, (**C**) Appressoria, and (**D**) Penetration. Different lowercase letter(s) above the bars in the same cuticle topography indicate statistically significant differences (Tukey’s HSD test; *P* < 0.05).

Under SEM, the shape of *C. fumosorosea* conidia was fusiform-elliptical. The two strains exhibited similarities in conidial size (i.e., length × width) with overall dimensions of 3.69 × 1.20 μM (for IFCF01) and 3.67 × 1.20 μm (for IFCF-D58) (Figure 5A). A high conidial germination rate (>90%) was obtained on all cuticular surfaces of *P. xylostella* larvae, and no significant difference was found between the two strains (*F_1,16_* = 1.888, *P* = 0.191) (Figure 5B). However, significant differences in the appressoria formation were noted between the strains. The IFCF01 strain was associated with a significantly higher appressoria rate (*F_1,16_* = 1639.16, *P* < 0.001) than IFCF-D58. The appressoria rates of IFCF01 at each surface topography, including gentle, spinous, and acanthoid, were 89.3 ± 2.31%, 92.0 ± 4.00%, and 89.3 ± 2.31%, respectively, and no significant difference was observed among these surfaces (*F_2,6_* = 0.800, *P* = 0.492). Furthermore, there were lower appressoria rates associated with IFCF-D58 strain at all surface topographies ranging between 10.67 ± 2.30% to 12.00 ± 4.00%, and no significant difference was observed among them (*F_2,6_* = 0.200, *P* = 0.824) (Figure 5C). The length of the penetrating hyphae of IFCF01 conidia was significantly shorter (*F_1,73_* = 196.60, *P* < 0.001) than IFCF-D58 on all surface topographies (Figure 5D). The longest penetrating hyphae in the case of IFCF01 were 2.54 ± 0.57, and 7.34 ± 1.28 μM in the case of IFCF-D58 were recorded in the gentle surface topography, while the shortest penetrating hyphae (0.73 ± 0.40 and 2.71 ± 0.96 μm in IFCF01 and IFCF-D58, respectively) were found in the acanthoid surface topography.

### 3.6. Colonization in the Hemocoel and Internal Tissues

**Figure 6:**
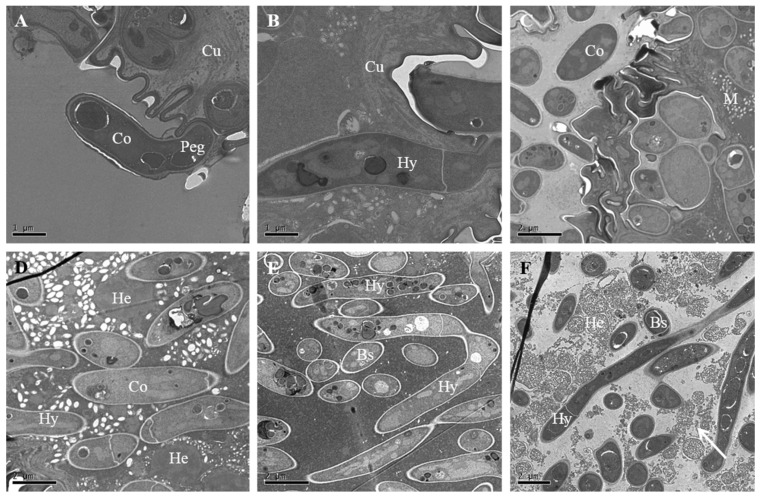
Stepwise invasion events of *Cordyceps fumosorosea* IFCF01 on the host integument visualized under transmission electron microscope. (**A**,**B**) were taken at 16 h after inoculation; (**A**) The tip of the conidia has differentiated into a penetration peg (Peg); (**B**) Hypha (Hy) has penetrated into the cuticular layer of the cuticle (Cu); (**C**) Hyphae have penetrated the cuticle and muscle (M) at 24 h after inoculation; (**D**,**E**) were taken 36 h after inoculation; (**D**) Hypha (Hy) and conidium (Co) have entered the muscle; hyphal bodies reproduced by created cell develop into blastospores (Bs). The hemocoele (He) has been colonized by blastospores; (**E**) Self-reproduction of the hyphal body (HB) was visible in the body cavity; (**F**) Hemocyte (He) damage (at arrow) was clearly visible, with disappearance of the nucleolus at 48 h after inoculation.

Observations recorded through TEM revealed that the conidia were initially attached to the epicuticle forming a depression on the host integument (Figure 6A), and their subsequent growth on the epicuticle loosen the procuticle (Figure 6B). The invading conidia, after passing through the procuticle, eventually penetrate the diamondback moth larval cuticle (Figure 6B) by damaging through the cuticular structure of diamondback moth larvae (Figure 6C). The conidia of the pathogenic fungus *C. fumosorosea* IFCF01, after passing through the integument, multiplies extensively in the hemocoel, and aggregates in the hemolymph of the host (Figure 6D,E). These yeast-like hyphal bodies are small spherical or rod-shaped hyphal fragments that proliferate through the hemocoel and infect and damage the larval internal tissues. This aggregation of blastospores within the hemocoel eventually help the invading pathogen to conquer the cellular defense mechanism of the *P. xylostella* larvae. At 48 h post-inoculation, hyphae massively self-reproduced by non-septa, one-septa, or two-septa means in the blood cavity, and the numbers of blastospores and hyphae in the hemocoel increased sharply, completely disrupting the hemocytes (Figure 6F).

### 3.7. Fungal Release from the Diamodback Moth Larval Cadavers

**Figure 7:**
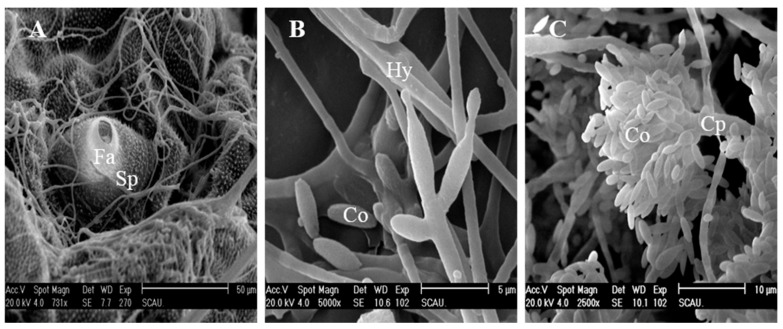
SEM photographs of conidia development of *Cordyceps fumosorosea* IFCF01 on the cuticle surface of *Plutella xylostella* larvae. (**A**) Development of hypha on the cuticle and around the spiracle (Sp) after 36 h post-inoculation. Fa = filter apparatus; (**B**) Hyphae developed, and differentiated to form a dense mycelial mass on the cuticle after 60 h post-inoculation; (**C**) Conidia development after 72 h post-inoculation, mature conidium (Co) is still attached to the conidiophores (Cp).

After consuming all the nutrients of the invaded host, the invading pathogen grows to cover the entire exoskeleton. Figure 7A clearly shows the colonies of conidia and the widespread network of germ tubes and well-develop mycelial mat colonizing the whole cuticular surface of the target host. Once hyphae extend to the exterior of the cadaver, they release large numbers of conidiophores. The phialides of the strain were characterized by a wide globose basal portion with a long distal neck (Figure 7C). The conidiophores were formed on the surface of larval cuticles at 60 h post-inoculation (Figure 7B). Many phialides with swollen bases and prominent necks were formed. Conidial chains elongated from the base as new conidia formed (Figure 7C). These conidia caused a secondary infection for other larvae in the surroundings.

## 4. Discussion

The successful attachment and germination of entomopathogenic fungi on the host epidermis is an important and critical step leading to the invasion of the host. The *P. xylostella* larvae treated with *C. fumosorosea* strain IFCF01 showed small brown melanism spots over their body surface. This indicated that the strain had penetrating points, which were advantageous for defeating the host’s defense system, and numerous penetrating points may be an important reason for why fungi can quickly invade target hosts. In our present study, conidia tended to adhere to surface areas containing protrusions (acanthoid and spinous surface topographies), and less to the gentle surface topography, and allowed us to speculate that the conidia of *C. fumosorosea* are sensitive to the cuticular surface topography.

In order to successfully invade the target host, attachment of fungal conidia to the host cuticle is very important [44]. In our study, the sclerous and smooth head capsule of the *P. xylostella* larvae prevented the attachment of the conidia of the strain of *C. fumosorosea* IFCF-D58. On the other hand, conidia and hyphae of another strain IFCF01 around the head capsule penetrated through the cuticle of *P. xylostella* larvae. These findings resonate with observations concerning the distribution of the conidia of widely studied *B. bassiana* on the head of the peach fruit moth [45]. Other studies have also demonstrated that conidia of the pathogenic fungus *Metarhizium anisopliae* produced infection structures when the germ tube contacted a hard surface [46]; i.e., the head capsule of *P. xylostella* was found to be vulnerable to *M. anisopliae* [39]. Generally, molting may serve as an insect defense that minimizes the colonisation ability of the conidia. In response, fungi must breach the cuticle, germinate and penetrate rapidly, and evade these behavioral responses. According to our results, strain IFCF01 could attach to, and invade, the head capsule, which implies that it could damage brain tissue, relatively quickly resulting in the destruction of the nervous system. This might be a reason why the strain IFCF01 exhibited higher pathogenicity against *P. xylostella*.

Dense acanthae on the thorax and abdomen of larvae should serve as a protective function for the insect. However, conidia of *C. fumosorosea* were trapped by, and tightly bound to, areas on the cuticle that contained small spines. On the one hand, these hair-like structures allow the larvae to walk on smooth and vertical surfaces; on the other hand, features of the acanthoid and spinous surface topography could favor the attachment and retention of fungal conidia. In our present study, *C. fumosorosea* strain IFCF01 produced specialized infection structures that included penetration pegs, appressoria, and growing hyphae to penetrate into the host integument. Furthermore, thorax and abdominal intersegmental folds are the primary sites where conidia began to start penetrating through the cuticle. Previous studies also reported similar observations, including *Metarhizium anisopliaeon* infecting *P. xylostella* [39] and the fungus *Erynia radicans* infecting *Empoasca fabae* [47]. Furthermore, conidia and hyphae of strain IFCF01 aggregated around and entered through the spiracles. Similar infection sites have also been found for the corn earworm *Heliothis zea* by *B. bassiana* [48]. In contrast, a previous study on *B. bassiana* found that conidia cannot enter the spiracles of longhorn beetles, *Apriona germari* [49], presumably because the filter apparatus in the spiracles serves to deter invasion.

According to the chronological events of the infection process, conidial germination, penetration, and colonization occur faster in strain IFCF01 compared with IFCF-D58, resulting in the earlier invasion of hosts infected by IFCF01. The two strains showed similarities in conidial size and germination rate, suggesting that it is not sufficient to screen for highly pathogenic strains based purely on these variables. On the other hand, a high proportion of malformed appressoria were found after inoculation with IFCF-D58, and conidia failed to penetrate host cuticles in infection, thus consuming its own-nutrients for further growth, and ultimately leading to the failure of the infection. The length of penetrating hyphae of strain IFCF01 was shorter than those of IFCF-D58 on all surface topographies, which suggested that the invasion speed of strain IFCF01 was relatively fast. Furthermore, we found that the acanthoid surface topography had significantly shorter penetrating hyphae than the other cuticle surfaces, suggesting a possible link between surface topography and invasion speed. This result coincided with external symptom observations of the infected larvae, with melanotic encapsulation presenting on the acanthoid surface topography in the early stage of invasion.

Limiting pathogens on the surface would imply that the immune system might represent more of a second and ultimately final effort at fighting the infection of certain pathogens than the cuticular defenses [44]. In the hemocoel of *P. xylostella* larvae infected with conidia of strain IFCF01, the blastospores subsequently infected the hemocytes and damage the cell membrane in addition to cytoplasm depletion. Fungal hyphae were detected in muscle fibers, and physical damage to internal tissues may have occurred due to the action of the blastospores and hyphae. A similar immune response has also been found in aphids where entry of hyphae into the hemocoel is followed by the formation of blastospores [50]. Once the host’s body nutrition was exhausted, *C. fumosorosea* IFCF01 passed through the surface of the cadaver to form new conidiophores that were then released and could produce new infections in other hosts. More mycelia were formed around the intersegments, which are relatively weak compared to other parts of the cuticle. Similar observations have been made in *B. bassiana*-infected *H. zea* [48] and in *B. bassiana*-infected *C. sasakii* [45].

Bioactive compounds from insect pathogenic fungi have already proven critical to the outcome of infection. Some studies have identified important secondary metabolites and cuticle-degrading enzymes that facilitate conidial penetration through the target host cuticle [51,52]. The current study revealed that a mucilage-like substance was secreted by strain IFCF01 when in contact with the host. It is possible that the mucilage is a matrix in which cuticle-degrading enzymes were active in penetrating the host cuticle. Previously, only a few studies have focused on the secretion of toxic proteins produced by *C. fumosorosea* during the infection process [19,53,54]. Our results revealed that larvae treated with strain IFCF01 did not exhibit morphological and cytoskeleton alterations similar to those seen in toxin-infected larvae, but showed a disruption, transformation, and disappearance of the regular parallel lamellar structure of the cuticle. An important implication of this result is that both the fungal mechanical force and hydrolytic enzymes may be involved in the infection. *P. xylostella* larvae mortality may be attributed to the mechanical disruption of the structural integrity of integuments by the growth of hyphae. Strain IFCF01 was capable of aggressive growth and propagation. Rapid production of blastospores and massive invasion in vivo may also play an important role in the infection process of *C. fumosorosea* to *P. xylostella*.

## 5. Conclusions

In conclusion, our study presents, for the first time, the infection events of *C. fumosorosea* against *P. xylostella* visualized under SEM and TEM. The infection process observed in this study revealed that following the attachment and germination of the fungal conidia of the pathogenic strain, penetration through the integument is by means of a specialized penetration peg. The yeast-like hyphal bodies within the host hemocoel damaged the internal tissues, overcoming the host defense mechanism and eventually leading to the death of the diamondback moth larvae. Further research should focus on the identification of important bioactive molecules, and to assess the production regulation of these bioactive molecules during the course of host infection by *C. fumosorosea* strain IFCF01.

## Data Availability

The data presented in this study are available on request from the corresponding author.

## References

[1] Talekar N.S., Shelton A.M. (1993). Biology, Ecology, and Management of the Diamondback Moth. Annu. Rev. Entomol..

[2] Soleymanzade A., Khorrami F., Forouzan M. (2019). Insecticide toxicity, synergism and resistance in *Plutella xylostella* (Lepidoptera: Plutellidae). Acta Phytopathol. Entomol. Hungarica.

[3] Zhang S., Zhang X., Shen J., Mao K., You H., Li J. (2016). Susceptibility of field populations of the diamondback moth, *Plutella xylostella*, to a selection of insecticides in Central China. Pestic. Biochem. Physiol..

[4] Kang W.J., Koo H.-N., Jeong D.-H., Kim H.K., Kim J., Kim G.-H. (2017). Functional and genetic characteristics of Chlorantraniliprole resistance in the diamondback moth, *Plutella xylostella* (Lepidoptera: Plutellidae). Entomol. Res..

[5] Wang X., Wang J., Cao X., Wang F., Yang Y., Wu S., Wu Y. (2019). Long-term monitoring and characterization of resistance to chlorfenapyr in *Plutella xylostella* (Lepidoptera: Plutellidae) from China. Pest Manag. Sci..

[6] Xu D., Ali S., Huang Z. (2011). Insecticidal activity influence of 20-Hydroxyecdysone on the pathogenicity of *Isaria fumosorosea* against *Plutella xylostella*. Biol. Control.

[7] Duarte R.T., Gonçalves K.C., Espinosa D.J.L., Moreira L.F., De Bortoli S.A., Humber R.A., Polanczyk R.A. (2016). Potential of entomopathogenic fungi as biological control agents of Diamondback Moth (Lepidoptera: Plutellidae) and compatibility with chemical insecticides. J. Econ. Entomol..

[8] AlJabr A., Hussain A., Rizwan-ul-haq M. (2018). Toxin-Pathogen synergy reshaping detoxification and antioxidant defense mechanism of *Oligonychus afrasiaticus* (McGregor). Molecules.

[9] Hussain A., AlJabr A.M. (2020). Potential synergy between spores of *Metarhizium anisopliae* and plant secondary metabolite, 1-chlorooctadecane for effective natural acaricide development. Molecules.

[10] Kepler R.M., Luangsa-ard J.J., Hywel-Jones N.L., Quandt C.A., Sung G.-H., Rehner S.A., Aime M.C., Henkel T.W., Sanjuan T., Zare R. (2017). A phylogenetically-based nomenclature for Cordycipitaceae (Hypocreales). IMA Fungus.

[11] Zimmermann G. (2008). The entomopathogenic fungi *Isaria farinosa* (formerly *Paecilomyces farinosus*) and the Isaria fumosorosea species complex (formerly *Paecilomyces fumosoroseus*): Biology, ecology and use in biological control. Biocontrol Sci. Technol..

[12] Lu L.H., He Y.R., Wu Y.J., Feng X., Chen H. (2007). The time–dose mortality model of a *Paecilomyces fumosoroseus* isolate on the diamondback moth, *Plutella xylostella*. Acta Entomol. Sin..

[13] Lei Y.Y., Lü L.H., He Y.R., Liang S.Y. (2010). Correlation between biological characteristics of *Isaria fumosorosea* and its pathogenicity against *Plutella xylostella*. Acta Phytopathol. Sin..

[14] Xie M., Wang L., Lu L., Zhao R., He Y. (2014). Characterisation of *Isaria fumosorosea* isolates and their virulence toward the Diamondback Moth, *Plutella xylostella*. Biocontrol Sci. Technol..

[15] Hussain A., Tian M.Y., He Y.R., Bland J.M., Gu W.X. (2010). Behavioral and electrophysiological responses of *Coptotermes formosanus* Shiraki towards entomopathogenic fungal volatiles. Biol. Control.

[16] Tian J., Diao H., Liang L., Hao C., Arthurs S., Ma R. (2015). Pathogenicity of *Isaria fumosorosea* to *Bemisia tabaci*, with some observations on the fungal infection process and host immune response. J. Invertebr. Pathol..

[17] Gao T., Wang Z., Huang Y., Keyhani N.O., Huang Z. (2017). Lack of resistance development in *Bemisia tabaci* to *Isaria fumosorosea* after multiple generations of selection. Sci. Rep..

[18] Avery P.B., Wekesa V.W., Hunter W.B., Hall D.G., McKenzie C.L., Osborne L.S., Powell C.A., Rogers M.E. (2011). Effects of the fungus *Isaria fumosorosea* (Hypocreales: Cordycipitaceae) on reduced feeding and mortality of the Asian citrus psyllid, *Diaphorina citri* (Hemiptera: Psyllidae). Biocontrol Sci. Technol..

[19] Keppanan R., Krutmuang P., Sivaperumal S., Hussain M., Bamisile B.S., Aguila L.C.R., Dash C.K., Wang L. (2019). Synthesis of mycotoxin protein IF8 by the entomopathogenic fungus *Isaria fumosorosea* and its toxic effect against adult *Diaphorina citri*. Int. J. Biol. Macromol..

[20] Huang W., Huang Y., Hao Y., Huang S., Gao T., Keyhani N.O., Huang Z. (2020). Host-dependent contributions of the Cfcdp1 protease gene to virulence in the entomopathogenic fungus *Cordyceps fumosorosea*. Pest Manag. Sci..

[21] Hussain A., Rizwan-ul-Haq M., Al-Ayedh H., AlJabr A. (2016). Susceptibility and immune defence mechanisms of *Rhynchophorus ferrugineus* (Olivier) (Coleoptera: Curculionidae) against entomopathogenic fungal infections. Int. J. Mol. Sci..

[22] Hussain A., Rizwan-ul-Haq M., AlJabr A.M., Al-Ayedh H. (2020). Evaluation of host–pathogen interactions for selection of entomopathogenic fungal isolates against *Oligonychus afrasiaticus* (McGregor). BioControl.

[23] Hussain A., Tian M.Y., He Y.R., Ahmed S. (2009). Entomopathogenic fungi disturbed the larval growth and feeding performance of *Ocinara varians* (Lepidoptera: Bombycidae) larvae. Insect Sci..

[24] Lei Y.Y., He Y.R., Lü L.H. (2011). Physiological defense responses of *Plutella xylostella* (Lepidoptera: Plutellidae) larvae infected by entomopathogenic fungus *Isaria fumosorosea*. Acta Entomol. Sin..

[25] Feldhaar H., Gross R. (2008). Immune reactions of insects on bacterial pathogens and mutualists. Microbes Infect..

[26] Kwon H., Bang K., Cho S. (2014). Characterization of the hemocytes in larvae of *Protaetia brevitarsis* seulensis: Involvement of Granulocyte-Mediated Phagocytosis. PLoS ONE.

[27] Valanne S., Wang J.-H., Rämet M. (2011). The Drosophila Toll Signaling Pathway. J. Immunol..

[28] Stokes B.A., Yadav S., Shokal U., Smith L.C., Eleftherianos I. (2015). Bacterial and fungal pattern recognition receptors in homologous innate signaling pathways of insects and mammals. Front. Microbiol..

[29] Hussain A., Ali M.W., AlJabr A.M., Al-Kahtani S.N. (2020). Insights into the *Gryllus bimaculatus* immune-related transcriptomic profiling to combat naturally invading pathogens. J. Fungi.

[30] Hussain A., Li Y.F., Cheng Y., Liu Y., Chen C.C., Wen S.Y. (2013). Immune-related transcriptome of *Coptotermes formosanus* Shiraki workers: The defense mechanism. PLoS ONE.

[31] Isaka M., Kittakoop P., Kirtikara K., Hywel-Jones N.L., Thebtaranonth Y. (2005). Bioactive Substances from Insect Pathogenic Fungi. Acc. Chem. Res..

[32] Qu S., Wang S. (2018). Interaction of entomopathogenic fungi with the host immune system. Dev. Comp. Immunol..

[33] Hussain A. (2018). Reprogramming the virulence: Insect defense molecules navigating the epigenetic landscape of *Metarhizium robertsii*. Virulence.

[34] Wang Q., Xu L. (2012). Beauvericin, a Bioactive Compound Produced by Fungi: A Short Review. Molecules.

[35] Zhang H., Hu W., Xiao M., Ou S., Hu Q. (2017). Destruxin A Induces and Binds HSPs in *Bombyx mori* Bm12 Cells. J. Agric. Food Chem..

[36] Mallebrera B., Prosperini A., Font G., Ruiz M.J. (2018). In vitro mechanisms of Beauvericin toxicity: A review. Food Chem. Toxicol..

[37] Weng Q., Zhang X., Chen W., Hu Q. (2019). Secondary Metabolites and the Risks of *Isaria fumosorosea* and *Isaria farinosa*. Molecules.

[38] Moino A., Alves S.B., Lopes R.B., Neves P.M.O.J., Pereira R.M., Vieira S.A. (2002). External development of the entomopathogenic fungi *Beauveria bassiana* and *Metarhizium anisopliae* in the subterranean termite *Heterotermes tenuis*. Sci. Agric..

[39] Wang Y., Lei Z.R., Zhang Q.W., Wen J. (2005). Microscopic observations of infection process of *Metarhizium anisopliae* on the cuticle of the diamondback moth, *Plutella xylostella*. Acta Entomol. Sin..

[40] Lei Y.Y., Lü L.H., He Y.R., Wei B. (2011). The symptoms and histopathological changes of *Plutella xylostella* larvae infected with *Isaria fumosorose*. Acta Phytopathol. Sin..

[41] Nian X., He Y., Lu L., Zhao R. (2015). Evaluation of alternative *Plutella xylostella* control by two *Isaria fumosorosea* conidial formulations—Oil-based formulation and wettable powder—Combined with *Bacillus thuringiensis*. Pest Manag. Sci..

[42] Nian X., He Y., Lu L., Zhao R. (2015). Evaluation of the time-concentration-mortality responses of *Plutella xylostella* larvae to the interaction of *Isaria fumosorosea* with the insecticides beta-cypermethrin and *Bacillus thuringiensis*. Pest Manag. Sci..

[43] Safavi S.A., Shah F.A., Pakdel A.K., Reza Rasoulian G., Bandani A.R., Butt T.M. (2007). Effect of nutrition on growth and virulence of the entomopathogenic fungus *Beauveria bassiana*. FEMS Microbiol. Lett..

[44] Ortiz-Urquiza A., Keyhani N. (2013). Action on the Surface: Entomopathogenic Fungi versus the Insect Cuticle. Insects.

[45] Xiong Q., Xie Y., Zhu Y., Xue J., Li J., Fan R. (2013). Morphological and ultrastructural characterization of *Carposina sasakii* larvae (Lepidoptera: Carposinidae) infected by *Beauveria bassiana* (Ascomycota: Hypocreales: Clavicipitaceae). Micron.

[46] St Leger R.J., Butt T.M., Goettel M.S., Staples R.C., Roberts D.W. (1989). Production in vitro of appressoria by the entomopathogenic fungus *Metarhizium anisopliae*. Exp. Mycol..

[47] Wraight S.P., Butt T.M., Galaini-Wraight S., Allee L.L., Soper R.S., Roberts D.W. (1990). Germination and infection processes of the entomophthoralean fungus *Erynia radicans* on the potato leafhopper, *Empoasca fabae*. J. Invertebr. Pathol..

[48] Pekrul S., Grula E.A. (1979). Mode of infection of the corn earworm (*Heliothis zea*) by *Beauveria bassiana* as revealed by scanning electron microscopy. J. Invertebr. Pathol..

[49] Wang X.H., Huang D.Z., Yang Z.Q., Li H.P., Zheng J.W. (2009). Microscopic observations of infection process of *Beauveria bassiana* on the cuticle of *Apriona germari* larvae. Sci. Seric..

[50] Askary H., Benhamou N., Brodeur J. (1999). Ultrastructural and Cytochemical Characterization of Aphid Invasion by the Hyphomycete *Verticillium lecanii*. J. Invertebr. Pathol..

[51] Safavi S. (2010). Isolation, Identification and Pathogenicity Assessment of a new Isolate of Entomopathogenic Fungus, *Beauveria bassiana* in Iran. J. Plant Prot. Res..

[52] Khan S., Nadir S., Lihua G., Xu J., Holmes K.A., Dewen Q. (2016). Identification and characterization of an insect toxin protein, Bb70p, from the entomopathogenic fungus, *Beauveria bassiana*, using *Galleria mellonella* as a model system. J. Invertebr. Pathol..

[53] Asaff A., Cerda-García-Rojas C., de la Torre M. (2005). Isolation of dipicolinic acid as an insecticidal toxin from *Paecilomyces fumosoroseus*. Appl. Microbiol. Biotechnol..

[54] Liu L., Zhang J., Chen C., Teng J., Wang C., Luo D. (2015). Structure and biosynthesis of fumosorinone, a new protein tyrosine phosphatase 1B inhibitor firstly isolated from the entomogenous fungus *Isaria fumosorosea*. Fungal Genet. Biol..

